# Navigating challenges and opportunities: a SWOT analysis of digital management center implementation in county hospitals

**DOI:** 10.3389/fpubh.2026.1767910

**Published:** 2026-02-09

**Authors:** Shouzhi Zhou, Hongqing Yin

**Affiliations:** Hospital Administrative Office, Affiliated Kunshan Hospital of Jiangsu University, Kunshan, China

**Keywords:** digital, digital healthcare, hospital management, informatization, SWOT analysis

## Abstract

**Objective:**

To comprehensively analyze the internal and external environmental factors influencing the construction of digital management center in county hospitals and to provide reference and basis for the construction and operation of digital management centers in county hospitals.

**Method:**

Employ the SWOT analysis to examine internal and external environmental factors influencing the development of digital management centers in county hospitals and propose policy reform recommendations.

**Results:**

Establishing digital management centers in county hospitals offers the advantage of consolidating dispersed hospital data, enabling unified management, real-time monitoring and information sharing. This initiative not only capitalizes on opportunities arising from the national health big data initiative but also overcomes disadvantages such as talent shortages, operational challenges and information silos, while mitigating threats like lagging hospital management.

**Conclusion:**

Following a comprehensive assessment and analysis of strengths, weaknesses, opportunities and threats, corresponding strategies and improvement measures have been formulated to advance the establishment of digital management centers in county hospitals. These initiatives provide theoretical support and policy recommendations for the practical implementation and operation of digital management systems within county hospitals.

## Highlights

- Targets the under-researched county hospital level in China’s healthcare reform, offering a structured SWOT analysis for digital intelligence center construction.
- Translates national “Healthy China” and “Digital China” strategies into actionable implementation pathways for grassroots hospitals.
- Identifies critical non-technical barriers-talent gaps, weak management, and data fragmentation-as central to successful adoption.
- Proposes context-specific strategic options to suit diverse institutional capacities.
- Positions digital centers beyond internal use-as core enablers for regional medical consortia and integrated care delivery.

## 1 Background

The high-quality development of the healthcare service system is a key component of the in-depth implementation of the “Healthy China” strategy and primary task in establishing a high-quality, efficient healthcare service system with Chinese characteristics (1, 2). In the new development stage, the existing healthcare service system can no longer meet the higher-level health needs of the people, particularly it faces a series of challenges such as extensive development focused on scale expansion, fragmented, disjointed healthcare services and outdated management methods, which severely hinder its high-quality development (3–6). Therefore, it is necessary to shift the development model of healthcare toward a greater emphasis on quality-driven growth, transform service delivery models to prioritize system-wide continuity, adopt management approaches focused on scientific governance. The aim of these reforms is to promote the expansion and decentralization of high-quality healthcare resources, achieving balanced regional distribution (7, 8). The rapid advancement of digital and intelligent technologies, coupled with the national strategies of “Healthy China” and “Digital China” provide strong momentum and significant opportunities for the high-quality development of the healthcare service system (9, 10). Establishing a comprehensive, high-quality, efficient healthcare service system in China and achieving high-quality development driven by digital technologies within this system, means focusing on cloud computing, big data technology, privacy computing, blockchain, Internet of Things, mobile internet and artificial intelligence as development engines. This approach aims to enhance the intrinsic efficiency of “development methods,” achieve high-quality and sustainable integration of “supply models,” drive the scientific modernization of “management methods.”Ultimately, it seeks to meet the people’s expectations for high-quality healthcare services, healthy and happy life. With the development of digital healthcare, construction of hospital digital centers has become increasingly important. Hospitals have already established information systems for performance management, cost management, price management, financial management and asset management. However, there are multiple technology suppliers and integration level of these systems is low, lacking unified management, resulting in severe “information silos” (11). Hospital information management systems lack standardized management processes in areas such as data acquisition pathways, statistical criteria, data quality control and data output. Additionally, insufficient data quality and value creation have led to challenges in the development and utilization of data resources (12). Therefore, hospitals urgently need to establish a comprehensive, convenient, intuitive integrated reporting center and data analysis platform for analysis and management, providing support for optimizing resources and promoting disciplinary development. In December 2020, the “Guiding Opinions on Strengthening the Operational Management of Public Hospitals” [National Health Commission Document (2020) No. 27] required hospitals to strengthen standardized operations and information integration platform construction, establish operational management systems and data centers, achieve full-process resource management (13). In 2021, General Office of the State Council issued the“Opinions on Promoting the High-Quality Development of Public Hospitals”which clearly proposed improving the level of refined management in hospitals, establishing a decision-support system based on data and using big data to improve hospital operational management levels. In 2021, General Office of the State Council issued the“Opinions on Promoting the High-Quality Development of Public Hospitals” clearly proposing to improve the level of refined management in hospitals, establish a data-driven hospital operational management decision-support system and use big data concepts to evaluate hospital disease combinations, cost outputs, hospital performance to improve efficiency and reduce costs (14). Establishing a digital management platform and applying big data technology to strengthen hospitals’ decision-making and analytical capabilities, thereby improving the precision of hospital management, is an important direction for digital hospital construction (15). From the perspective of hospital development, hospital business activities, economic activities and resource allocation activities are becoming increasingly complex. There is an urgent need to address shortcomings in internal operational management while maintaining public welfare and to seek efficiency gains through precision management. From the perspective of hospital practice, the informatization of operational management in public hospitals generally faces issues such as low data standardization, weak system integration capabilities and insufficient functional expansion (16, 17). Meanwhile, hospital management has placed higher demands on the information platform’s human-machine interaction capabilities, intelligent analysis and monitoring capabilities, service extension capabilities, providing robust support for the construction of integrated information systems within county medical alliances. An digital platform is a crucial tool for driving the high-quality development of public hospitals (18). Therefore, digital technology will become the core engine for the high-quality development of the healthcare service system, driving a transformation in service models from “scale expansion” to “value-based healthcare,” achieving a win-win outcome of improved patient satisfaction and reduced operational costs. Additionally, establishing a digital driven management center for county-level hospitals can provide a data engine for the high-quality development of individual hospitals while laying the technological foundation for medical consortium construction, ultimately achieving the transformation of the county healthcare service system from “fragmented” to “integrated.”This paper employs the SWOT strategic analysis tool to comprehensively consider and systematically analyze the internal and external environmental factors influencing the construction of digital management centers in county hospitals, providing theoretical support and policy recommendations for the construction and operation of such centers.

## 2 SWOT analysis

SWOT Analysis (short for strengths, weaknesses, opportunities, threats) is a business strategy tool to assess how an organization compares to its competition (19). SWOT analysis is adynamic analysis method that can take into account the overall strategic analysis, which is widely used in artificial intelligence (20), Robotic technology (21), nursing workforce policy (22), community health nursing (23), medical equipment (24), Healthcare health care (25)and other medical fields are widely utilized.

## 3 Methods

### 3.1 Advantages, disadvantages, opportunities, and challenges of establishing a digital management center in county hospitals

#### 3.1.1 Advantage (S)

##### 3.1.1.1 Unified management

The diversity of healthcare information systems, the variety of multi-channel data sources and the multi-point statistical standards result in inconsistent data formats. This prevents real-time data monitoring, hinders the full realization of big data’s benefits and consequently poses challenges for big data analytics (26). To meet the demands of hospital management for data utilization, hospital management and information technology assessment standards, disciplinary development and scientific research management needs, as well as regional data interoperability requirements, hospitals urgently need to establish an efficient digital management center. Hospital management has recognized the importance of the comprehensive quality management model. Decision-makers have adopted innovative approaches to integrate resources and coordinate cross-departmental efforts, actively advancing the establishment of an digital management center. This center employs appropriate analytical methods to conduct precise analysis of hospital-wide big data, establishing an efficient and unified quality management data decision-making repository.

##### 3.1.1.2 Real-time tracking

The hospital digital management center serves hospital administration and is applied in clinical settings to meet the diverse service needs of medical personnel at various levels. The data management center must ensure comprehensive coordination, controlling the timeliness, accuracy and continuity of data. It efficiently utilizes data resources to assist departments and decision-makers in formulating management strategies, continuously optimizing the operational mechanisms of the quality management data center (27). The hospital digital management center leverages the hospital-wide network system platform to integrate dispersed functional department business systems. These include the outpatient and emergency information center, outpatient appointment center, clinical data center, five centers (chest pain center, trauma center, stroke center, critical maternal care center, critical pediatric and neonatal care center), performance center, operations center, medical quality management center, research data center and logistics management center. The system supports real-time querying and monitoring of relevant data indicators across the entire hospital. It enables precise analysis through dimensions such as hospital tiered evaluation metrics, national or provincial quality control indicators and hospital “15th Five-Year Plan” development targets. This facilitates data-driven digital management, highlighting the critical importance of real-time data monitoring.

##### 3.1.1.3 Information sharing

During the 14th Five-Year Plan period, China’s digital economy has entered a new phase characterized by inclusive sharing and other functions. The development of data-driven healthcare faces new demands and expectations. Utilizing digital technologies to accelerate the integration of regional healthcare services is the future trend of China’s digital healthcare development (28). Under the backdrop of hospitals entering an era of big data and high-quality development, institutions should establish digital management centers to integrate internal data sources, unlock data value and transform latent data resources into an internal driving force for hospital advancement. This initiative aims to pursue high-quality, high efficiency hospital development, achieve data sharing capabilities and lay the foundation for building a digital hospital (29). By establishing a digital management center, hospitals can integrate clinical, operational, research data centers to systematically organize and consolidate data spanning clinical diagnosis and treatment, operational management, performance evaluation, personnel management, quality control and research projects, thereby implementing quality control management. This enables the full utilization of big data resources to meet the needs of hospital administration, clinical research, performance management and talent development. Functional departments can leverage the data-sharing capabilities of the digital management center to achieve benefits such as clinical decision support and the generation of business analysis reports, thereby driving reform and development in the precise application of big data.

#### 3.1.2 Weakness (W)

##### 3.1.2.1 Talent bottleneck

The core talent bottleneck in the construction of county hospital digital management centers primarily manifests as dual deficiencies in technical capabilities and team structure. Due to the diversity and heterogeneity of information systems, IT departments often lack the technical capabilities required to maintain complex systems, making it difficult to ensure data integration and system stability. At the same time, operational departments lack the ability to apply data analytics tools, hindering their capacity to fully unlock the value of healthcare data. The information department lacks a dedicated operations and maintenance team, while quality management positions lack professionals with both medical knowledge and data analysis capabilities, making it difficult to implement data-driven quality control management. The dual constraints of technological gaps and shortages of specialized talent directly hinder the development of digital management centers. This makes standardized governance of cross-system data difficult to achieve and further impedes the support of digital applications such as clinical decision-making and performance management. The fundamental contradiction lies in the mismatch between resource endowment of county medical institutions and high technical demands of digital development. This issue urgently requires resolution through multiple channels, including targeted training, external recruitment and industry-academia-research collaboration.

##### 3.1.2.2 Operational challenges

The operational challenges in establishing a digital management center in county hospitals are primarily manifested in systemic deficiencies in cross-departmental collaboration and data governance. From a management structure perspective, blurred boundaries between multiple functional departments have led to fragmented data management processes, resulting in a situation where data is managed by many parties yet no one is held accountable. At the data level, the absence of unified data standards and governance mechanisms has led to inconsistent data formats across systems. This has triggered issues such as redundant data collection and uneven data quality, significantly increasing the error rate in critical indicators like healthcare quality monitoring data. The phenomenon of data silos further exacerbates two core contradictions: operational departments struggle to access real-time, accurate integrated data, leading to delays in precision management tasks such as DRG cost accounting;quality management departments are unable to establish closed-loop management systems, prolonging the response cycle from issue identification to corrective action. The deeper issue lies in the fact that the current management model has yet to establish an integrated mechanism spanning “systems-data-applications, “making it difficult to implement the policy-mandated transformation toward intelligent quality control. These structural deficiencies place county hospitals at a strategic disadvantage when confronting policy changes such as medical insurance payment reforms and performance evaluations, due to insufficient data support.

##### 3.1.2.3 Information silos

The heterogeneity of hospital business system databases is particularly pronounced. The development of each business system has only addressed the needs of its respective department, lacking long-term, forward-looking and systematic planning. The current lack of unified technical and data standards, the inability to automatically transmit data, the absence of effective linkage and sharing mechanisms have led to the formation of numerous “information silos” within hospitals. Certain metrics are difficult to obtain, data suffers from non-standardization issues and there is a lack of support from a hospital-wide knowledge base (30–37). The core reason behind the information silo problem encountered lies in the lack of systematic planning and data governance during the construction of digital management centers in county-level hospitals. The core issue lies in the adoption of heterogeneous database architectures across various business systems (HIS, LIS, PACS, etc.). Their development has focused solely on departmental needs without hospital-wide coordination, resulting in inconsistent data standards and a lack of standardized interfaces. This fragmented construction model triggers a three fold chain reaction: (1) Data flow is obstructed, preventing the automatic exchange of critical operational data such as clinical diagnoses and medication inventory, necessitating manual re-entry. (2) Data value is compromised, as structured data like electronic medical records and diagnostic tests cannot be cross-system linked, hindering advanced applications like Diagnosis-Related Group (DRG) cost analysis. (3) The absence of a unified data middleware platform leads to inefficient collection of real-world data (RWD) required for research, thereby constraining disciplinary advancement. These issues highlight that county hospitals have yet to establish a collaborative mechanism encompassing “top-level design, standard formulation and system restructuring” during their digital transformation process. There is an urgent need to break down data silos through technical means such as Master Data Management (MDM).

#### 3.1.3 Opportunity (O)

##### 3.1.3.1 National policy support

The “National Health and Medical Big Data Standards, Security, and Service Management Measures (Trial)” [National Health Commission Planning, Development, and Informatization Department Document (2018) No. 23], “Notice on Conducting the ‘Public Medical Institutions Economic Management Year’ Activity” [National Health Commission Financial Affairs Department Letter (2020) No. 262] and other documents elevate the development of health and medical big data to a national strategy. Through strategic planning, these policies promote the construction of a national integrated data center collaborative innovation system, with a focus on regions such as Beijing-Tianjin-Hebei and Yangtze River Delta as demonstration areas. This strengthens the layout of county hospital digital management centers and their integration with regional core node functions. Special funds for public hospital reforms are being allocated to county digital platforms to support the construction of smart hospitals. This policy framework, guided by strategic planning and supported by fiscal resources, systematically addresses persistent challenges in county hospitals, such as data silos and inconsistent standards by advancing the establishment of digital management centers. It enhances the utilization of clinical data, positioning these centers as the core engine driving the digital transformation of county-level healthcare.

##### 3.1.3.2 Service advantages

As people’s demands for healthcare service quality continue to rise, hospitals need to enhance operational efficiency and social service capabilities through digital hospital construction to better meet the needs of patients. The maturity and development of technologies such as big data, artificial intelligence and Internet of Things provide technical support for hospital operations management and intelligence hospital construction, enabling more efficient data processing, more precise decision-making support and more intelligent equipment management. Through intelligence upgrades, hospitals can optimize resource allocation and achieve more refined management, thereby reducing costs related to human resources, materials, finances and improving both economic and social benefits. Additionally, hospital electronic health records are evolving toward intelligent and structured formats, not only recording patient diagnostic information but also automatically generating treatment recommendations, warning of medical risks and supporting the extraction of clinical research data, thereby serving as intelligent assistants throughout the entire patient care process. Patients can conveniently access medical information, test reports, and consultation services through mobile APP, WeChat and other mobile platforms, enabling them to book appointments, make payments and consult online, thereby enhancing their medical experience and satisfaction. Digital transformation will enable hospitals to break through traditional spatial and temporal constraints, establish an integrated online and offline service ecosystem, provide patients with more efficient, convenient and personalized medical services.

##### 3.1.3.3 Development needs

Hospital Information Systems (HIS), Laboratory Information Management Systems (LIS) and Picture Archiving & Communication Systems (PACS) will accelerate their migration to the cloud, enabling centralized data storage, real-time sharing and efficient management. Leveraging the powerful computing power and elastic scalability of cloud computing, hospitals can quickly process massive amounts of medical data, providing strong support for clinical decision-making and research innovation. The widespread adoption of medical IoT enables various medical devices, wearable devices and sensors to collect patients’ vital signs and diagnostic data, creating a “digital twin” of the patient. This enables comprehensive and dynamic monitoring of patients’ health conditions, laying the foundation for personalized and precision medicine. The deep integration of big data and artificial intelligence technologies unlocks the latent value of medical data, supporting hospitals in achieving refined management. Through in-depth analysis of clinical data, operational data and medical insurance data, hospitals can optimize resource allocation, reasonably arrange treatment processes, precisely control medical costs and improve operational efficiency. Artificial intelligence-assisted diagnosis systems can quickly and accurately identify disease imaging characteristics and assist doctors in formulating treatment plans, thereby improving diagnostic accuracy and treatment outcomes. Artificial intelligence empowers hospital logistics management, pharmaceutical management and equipment maintenance, achieving intelligent operations and maintenance, reducing labor costs and enhancing the overall operational efficiency of hospitals.

#### 3.1.4 Threat (T)

##### 3.1.4.1 Loose management

Common issues in China’s hospital quality management include inconsistent quality control standards, arbitrary performance evaluations, ineffective implementation of regulations, insufficient training intensity and a shortage of dedicated quality control personnel. These challenges are driving the transformation and upgrading of healthcare quality management (38). Weak data governance foundations lead to non-standardized data collection, missing key fields and frequent logical errors, significantly reducing data usability. Inadequate resource integration capabilities have led to a proliferation of heterogeneous systems within county institutions. Due to the absence of comprehensive design, imaging and laboratory data are stored in isolation, creating information silos. Technical and talent shortages hinder the advancement of intelligent systems, budget constraints limit the deployment of smart quality control tools, data cleansing relies heavily on manual operations and the severe shortage of professionals with expertise in both healthcare and information technology significantly restricts the depth of data value extraction. Heightened security risks manifest in unclear permission hierarchies and the absence of log auditing mechanisms. Limited security investments at county hospitals struggle to address legal disputes arising from data breaches or compliance failures. These issues collectively impede the development of digital management centers, severely undermining the operational effectiveness of hospital digital hubs.

##### 3.1.4.2 Data sharing

The data sharing challenges encountered primarily manifest in three aspects during the construction of a digital management center for county hospitals: (1)Due to the lack of unified planning in information system development, critical data such as laboratory test results, imaging materials and medical records are scattered across different platforms, creating “information silos.” (2)Inconsistent data collection standards, with different departments employing varying quality control metrics and data entry specifications, result in poor data compatibility and inefficient sharing. (3)The absence of effective data integration mechanisms hinders cross-departmental data exchange and operational collaboration, compromising both the timeliness and accuracy of clinical decision-making while obstructing the establishment of a closed-loop quality management system. These issues directly result in diminished operational efficiency and increased management costs, severely limiting the value realization of the digital management center. To address these challenges, it is imperative to establish unified data standards and a shared platform to dismantle information silos, enabling seamless data interoperability and efficient utilization.

## 4 Strategy

### 4.1 Strengths-opportunities strategy

It is imperative to seize external opportunities to establish a digital management center while simultaneously developing internal strengths. The greatest value in building a hospital digital management center lies in standardization, unification and ensuring the reliability of data sources for quality control. It emphasizes the role of quality control data in driving improvements in clinical business quality and enhancing the efficiency of functional department management, thereby realizing the value of data assets. Through data analysis, provide decision support for hospital leadership. Establishing a hospital digital management center enables administrators to access real-time, accurate and reliable data from clinical frontlines with ease, effectively achieving real-time data monitoring, data resource sharing and unified quality management. This provides a basis for making scientific decisions to drive the development of hospitals and promotes the gradual achievement of high quality development in public hospitals. By establishing a digital management center, hospitals can integrate data resources, optimize the allocation of medical resources, promote precise analysis, leverage the value of big data and implement multiple measures to expand big data applications. This will advance the standardization process of hospital quality management, ultimately achieving the goal of high-quality hospital development.

### 4.2 Weaknesses-opportunities strategy

In establishing a digital management center, it is essential to fully leverage external opportunities while effectively addressing internal shortcomings. Hospitals should actively respond to national policy support initiatives and establish digital management centers aligned with their own developmental positioning. This enables seamless, real-time and comprehensive management of quality control data, supporting cross-departmental sharing to ensure standardization, normalization and homogeneity. This initiative fully activates the latent value inherent in quality management data. The hospital’s digital management center provides services for hospital administration. Hospital administrators must prioritize establishing a quality management data center, promote the orderly implementation of all related tasks, ensure unified sources and consistent statistical standards for quality control data and guarantee compatibility of information data interfaces. This will maximize the value of quality control data, providing robust support for hospital decision-making and development.

### 4.3 Strengths-threats strategy

It is essential to fully leverage the internal strengths of establishing a digital management center while effectively identifying and mitigating external threats. Aligning with the trends in healthcare big data and considering the hospital’s actual circumstances, the business system resources of departments such as the outpatient and emergency information center, outpatient appointment center, clinical data center, five centers, performance evaluation center, quality management center and logistics management center should be integrated to establish a hospital digital management center. This center should focus on cultivating a multidisciplinary team proficient in hospital management, financial analysis, information technology, quality control and data analytics. By employing innovative thinking and methodologies, it should deeply mine data value, resolve issues of fragmented and inconsistent quality control data sources, ensure the authenticity and reliability of quality control data, achieve traceability of quality control data, facilitate cross-departmental or cross-regional data sharing and realize standardized quality control management across the entire hospital and even the entire region.

### 4.4 Weaknesses-threats strategy

It is necessary to overcome the inherent disadvantages of constructing a digital management center and avoid and mitigate external threats. The multi-source collection, duplicate input, multi-use and maintenance of hospital data information, coupled with poor synchronization in information updates, affect data consistency and accuracy. This results in a mixed source of hospital information resources, low efficiency in the utilization of information value, and a lack of unified, unique and reliable data attributes. Establish a hospital big data sharing platform between hospital’s digital management center and existing information system architecture, achieving data integration across multiple modules and systems, standardizing data standards, defining data usage rights and responsibilities. Expand the dimensions and scope of data usage and conduct multi-level data analysis. Strengthen the cultivation of specialized talent for the quality management data center, increase funding for specialized talent development, cultivate composite data processing, analysis talent through multiple channels, directions and positions to meet the hospital’s big data processing needs. Develop a dedicated plan for the quality management data center, utilize data analytics to evaluate business operations, support decision-making, optimize business processes and achieve coordinated development across business, quality, information and management. Integrate this initiative into the hospital’s overall information technology development strategy.

## 5 Conclusion

While ensuring data security, we should harness the vitality of digital grounded in algorithmic fairness and high computational power to serve as the core hub for the circulation and interaction of other production factors, the connecting link for fragmented healthcare services, the capability guarantee for modernizing hospital and government governance. Digital technology-driven high quality development of the healthcare service system supports the implementation of a health-first development strategy, promotes the integration of health into all public policies and encourages all regions and departments to prioritize safeguarding public health as a key objective of socioeconomic policies. Aiming to provide the public with more high-quality, efficient, sustainable, and equitable healthcare services, we will establish a healthcare service system with Chinese characteristics that is efficient and high-quality, inspiring public confidence and ensuring comfort and peace of mind. The construction of a digital management center for county-level hospitals facilitates the integration of clinical, operational, research data resources across the board, establishes a unified data governance system and provides a core driving force for the high-quality development of hospitals-internally achieving refined decision-making, closed-loop quality control, intelligent service upgrades and promoting the transition of hospitals from scale expansion to intrinsic development. Externally, it will serve as a digital hub for county medical consortium construction, breaking down “information silo” barriers, enabling real-time sharing of medical data such as laboratory tests and imaging, supporting the implementation of tiered diagnosis and treatment and medical insurance cost control, promoting the comprehensive allocation of resources and standardized management within medical consortia. Ultimately, the center plays a dual role as a “technical foundation and data link” empowering individual hospitals to achieve the high-quality development goals of “cost reduction, quality improvement and efficiency enhancement” while also providing core support for the construction of an integrated medical and health service system in the county that features “business coordination, resource integration and service continuity” achieving a two-way empowerment of hospital upgrades and regional medical coordination.

## 6 Limitations

Methodologically, the analysis relies solely on SWOT qualitative analysis, lacking quantitative data to support the universality of conclusions and fails to incorporate successful or failed case studies, thereby weakening the practical reference value of the strategies. This study focuses on macro-level factors but lacks sufficient analysis of regional disparities in county hospitals’ economic conditions and medical insurance policies, thereby limiting the adaptability of strategic implementation. It overlooks the digital collaboration mechanisms between county medical alliances and higher-level hospitals, as well as data interoperability bottlenecks within the tiered diagnosis and treatment system. The dynamic dimension fails to adequately explore the impact of AI technology iterations on the architecture of digital centers and does not incorporate newly enacted data element policies from 2024, resulting in limited research timeliness. Although the analysis of implementation barriers mentions talent shortages, it fails to propose concrete solutions. Data security measures are also inadequately elaborated. Furthermore, the absence of stakeholder perspectives and failure to investigate the needs of healthcare providers, patients and medical insurance departments undermines the “demand-driven” development logic. Moving forward, a phased and differentiated implementation approach should be established by integrating empirical data, dynamic policy adjustments and technological advancements.

## Data Availability

The original contributions presented in the study are included in the article/supplementary material, further inquiries can be directed to the corresponding author/s.
